# Clinical Characteristics of Methanol-Induced Optic Neuropathy: Correlation between Aetiology and Clinical Findings

**DOI:** 10.1155/2022/4671671

**Published:** 2022-11-09

**Authors:** Qiao Sun, Mingming Sun, Yuan Zhang, Song Wang, Wenhao Bai, Shihui Wei, Quangang Xu, Huanfen Zhou

**Affiliations:** ^1^Department of Ophthalmology, The Third Medical Center of Chinese PLA General Hospital & Chinese PLA Medical School, Beijing 100853, China; ^2^Department of Ophthalmology, Shanghai Aier Eye Hospital, No. 83 Wuzhong Road, Xuhui District, Shanghai 200235, China; ^3^Department of Ophthalmology, The First Medical Center of Chinese PLA General Hospital, Beijing 100853, China; ^4^Department of Ophthalmology, The Second Affiliated Hospital of Anhui Medical University, Hefei 230601, China

## Abstract

**Purpose:**

To show the clinical characteristics, identify the magnetic resonance imaging (MRI) and optical coherence tomography (OCT) features, and observe the visual outcome of methanol-induced optic neuropathy.

**Methods:**

Clinical data were retrospectively collected from in-patients diagnosed with methanol-induced optic neuropathy in the Neuro-Ophthalmology Department of the Chinese People's Liberation Army General Hospital from January 2016 to January 2021.

**Results:**

Eight patients were included in this study. The exposure time was 6–34 h for ingestion, 3-4 months for inhalation, and more than ten years for skin absorption. All patients demonstrated bilateral acute visual impairment. Seven of eight patients had other accompanying systemic symptoms. Seven of eight patients demonstrated optic nerve lesions in MRI, and five presented with a hyperintense T2 signal in a “central” type. OCT showed the macular ganglion cell layer and inner plexiform layer (mGCL-IPL) thinning before the peripapillary retinal nerve fiber layer (pRNFL) thinning. The visual improvement was achieved transiently for seven of eight patients after treatment. One patient with a mitochondrial DNA mutation maintained a bilateral no-light perception (NLP) from the onset to the last visit. All patients had poor visual prognoses, with either light perception or NLP.

**Conclusions:**

Methanol-induced optic neuropathy is a rare bilateral optic neuropathy with a poor visual outcome. A centrally hyperintense T2 signal of the optic nerve is common in methanol-induced optic neuropathy. The thinning of the mGCL-IPL is more sensitive than that of the pRNFL for early diagnosis. A mitochondrial genetic defect may be a predisposing factor for methanol-induced optic neuropathy.

## 1. Introduction

Methanol poisoning is a serious life-threatening condition with a mortality rate ranging from 18 to 44% [1]. Persistent visual sequelae are seen in 30–40% of survivors [2]. During the COVID-19 pandemic, the methanol poisoning rate increased unexpectedly in Iran [3, 4], Turkey [5], and the United States [6, 7]. Most patients were misled by the misinformation that ingesting alcohol or hand sanitizers could protect against COVID-19, while some were caused by alcohol abuse associated with fear and anxiety. This resulted in 800 deaths, and many survived with blindness or visual impairment [3–7]. Thus, methanol poisoning continues to be an important issue in public health, emergency, and ophthalmology worldwide.

Methanol is a volatile watery liquid with a pungent alcohol odor. It is naturally contained in fermented beverages and widely used in industry, including cleaning products, antifreeze, pesticides, and fuel sources [8, 9]. Accidental oral ingestion is the primary exposure route [1, 3, 9–11]. Inhalation and dermal absorption associated with occupational exposure or intentional abuse have been increasingly reported [8, 12–19]. Methanol intoxication is caused by its metabolite formate, an inhibitory enzyme of the mitochondrial respiratory chain. This resulted in a decreased aerobic production of adenosine triphosphate (ATP) and the impairment of ATP-requiring intracellular reactions, which leads to systemic histotoxic hypoxia and metabolic acidosis [20].

The optic nerve, which is highly energy dependent, is vulnerable to mitochondrial dysfunction, including methanol poisoning. ATP shortage leads to the interruption of axoplasmic flow and intra-axonal swelling [21]. The papillomacular bundle fibers, which are small caliber axons rich in mitochondria, are particularly involved [22]. Therefore, methanol-induced optic neuropathy belongs to acquired mitochondrial optic neuropathy, although other mechanisms, including oxidative stress and proinflammatory cytokines, are also reported. Optic nerve histopathology showed mild edematous changes [23] and degeneration of axons and glial cells [2]. Demyelination or axonal necrosis changes are inconsistent in different studies [23, 24]. Accompanying damage to the retina may involve all its layers [2].

Methanol-induced optic neuropathy is rare in ophthalmic clinical practice. Patients usually come with mild or recovered systemic manifestations and an insidious exposure history, making it challenging to obtain an accurate diagnosis. Some studies reported that magnetic resonance imaging (MRI) abnormalities like T1 enhancement and/or long T2 in the retrobulbar segment of optic nerves were found in methanol-induced optic neuropathy [12, 25, 26]. Optical coherence tomography (OCT) finding of ganglion cell layer (GCL) loss selectively involving the papillomacular bundle was also reported [22]. In a four-year follow-up study, methanol-induced optic neuropathy showed progressive chronic axonal loss demonstrated by a depression of the N1P1 amplitude [27].

So far, several questions are worthwhile to be illuminated to advance our understanding. First, do morbidity and the prognosis of visual impairment differ according to the exposure route? Second, as magnetic resonance imaging (MRI) and optical coherence tomography (OCT) are objective and sensitive imaging modes for the optic nerve, can we obtain clues from them to assist in decision-making? Third, do mitochondrial gene mutations and nutritional status play a role in methanol-induced optic neuropathy?

We conducted this study to interpret the clinical characteristics of methanol-induced optic neuropathy in a Chinese cohort. The possible differences in latency, symptom, and visual prognosis caused by exposure variation will be discussed. MRI and OCT features will be given special attention.

## 2. Materials and Methods

### 2.1. Design and Study Population

This is a retrospective cross-sectional single-center study approved by the institutional review board of the Chinese People's Liberation Army General Hospital (PLAGH) and performed in accordance with the Helsinki Declaration. The clinical records of in-patients diagnosed with methanol-induced optic neuropathy from January 2016 to January 2021 with a follow-up period of over 6 months were collected from the PLAGH database. An experienced ophthalmologist-in-charge kept an additional telephone follow-up. Informed consent was obtained from all patients.

The inclusion criteria were as follows: (1) methanol is the primary exposure; (2) methanol is detected in the blood or urine; and (3) optic neuropathy is confirmed by the acute loss of visual acuity or visual field, a pupillary defect with light, and visual electrophysiology examination [8, 12].

The exclusion criteria were as follows: (1) optic neuropathy caused by inflammatory demyelination, vascular, infection, inflammation, compressive, or infiltrative causes; (2) optic neuropathy caused by other neurotoxic material, including drugs (e.g., ethambutol), metals (e.g., lead, mercury, and thallium), organic solvents (e.g., ethylene glycol, toluene, styrene, and perchloroethylene), and carbon dioxide; and (3) accompanied by other ocular diseases, including anterior segment, retinal, macular, and glaucoma.

### 2.2. Ophthalmological and Associated Examinations

Ophthalmological examinations included pupil examination, relative afferent pupillary defect testing, slit-lamp examination, and ophthalmic fundus examination. Best-corrected visual acuity (BCVA) was tested using the Snellen chart. BCVA below 0.01 was classified using the semiquantitative scales “finger count,” “hand motion,” “light perception (LP),” and “no light perception (NLP).” The standard protocols for the optic disc cube 200 × 200 circle scan and the macular cube 512 × 128 scan were performed using the spectral-domain OCT device (Carl Zeiss Meditec, USA). To be included, all scans had to have a signal strength ≥6 without motion artifacts. The optic nerve and brain MRI were imaged using a 3.0T scanner. The MRI protocols included T1-weighted image (T1WI) and T2-weighted image (T2WI) sequences with fat suppression before and after gadolinium administration.

### 2.3. Laboratory Tests

Blood tests were performed, including a complete blood count, serum chemistry panel, infectious disease tests (syphilis included), autoimmune tests (antinuclear antibody titer and anti-double-stranded DNA), and serum folic acid and vitamin B12 level tests.

Toxicological tests of blood and urine were performed in the acute phase. Mt-DNA mutation tests were arranged if consent was given.

### 2.4. Statistical Analysis

SPSS software package version 25 (SPSS Inc., Chicago, IL, USA) was used for statistical analysis. To test the normality of the distribution, the Kolmogorov–Smirnov test and the Shapiro–Wilk test were used. The level of statistical significance was *p* < 0.05. Quantitative variables with normal distribution were presented as means with standard deviation and ranges. Categorical variables were described as numbers and percentages.

## 3. Results

### 3.1. General Information and Exposure

The clinical characteristics are shown in Table 1. Eight sporadic patients (five male and three female) were included in the study, with a mean age of 48.4 ± 10.1 years (31–62 years). From onset to admission to the Neuro-Ophthalmology Department, the average presentation time was 56.1 ± 33.1 days (18 to 109 days). All patients had a mean follow-up time of 25.15 ± 14.10 months (8.6–42.6 months).

As shown in Table 1, five of the patients exposed through ingestion were male, with patients 1 and 2 drinking industry alcohol by mistake and patients 3, 4, and 5 accidentally drinking draught or counterfeit spirits. Two patients exposed through inhalation were female and were exposed in the workplace. Patient 6 worked in a solid alcohol factory. Patient 7, a barrel cleaner, used industry alcohol as a detergent. She wore gloves but not a mask while at work. Patient 8 was exposed through dermal absorption. She worked in a pipeline and constantly used industry alcohol as a detergent to clean her hands. The latency from exposure to onset was 6–34 h for ingestion, 3-4 months for inhalation, and more than 10 years for skin absorption (Table 1).

Serum folic acid and vitamin B12 levels were normal for all patients. As shown in Table 1, four patients underwent mitochondrial DNA mutation tests. Mutation m.14502 *T* > *C* was detected in one patient (patient 8).

### 3.2. Acute Symptoms

Only one patient in the ingestion group (patient 1) presented no obvious systemic symptoms. Among the patients, 87.5% (7/8) showed various acute systemic symptoms, including gastrointestinal, cardiopulmonary, renal, and encephalic disorders, and 25% (2/8) with comas received dialysis in the acute phase. One patient (patient 4) had an acute auditory impairment but he recovered six months later. No patient had a central nervous system complaint during their last visit, although two (patients 4 and 7) had a putaminal lesion in the brain MRI (Table 2).

### 3.3. MRI and OCT Features

**Figure 1:**
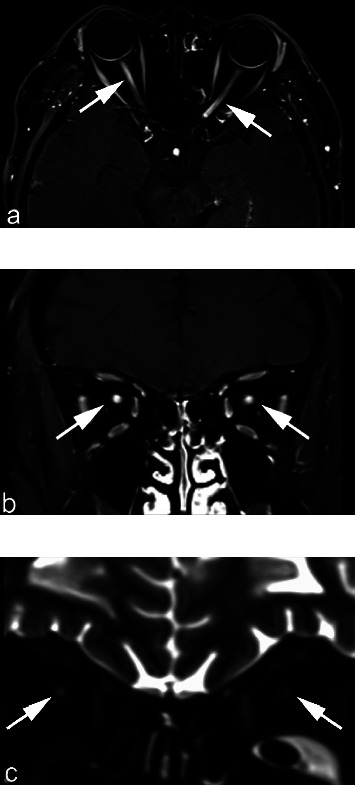
MRI identified optic nerve lesions in patients with methanol-induced optic neuropathy. Axial (a) and coronal (b) T1 enhancement images of patient 4, demonstrating hyperintense signals (arrows) in both optic nerves; (c) coronal T2 image of patient 8, demonstrating hyperintense signals (arrows) in both optic nerves.

**Figure 2:**
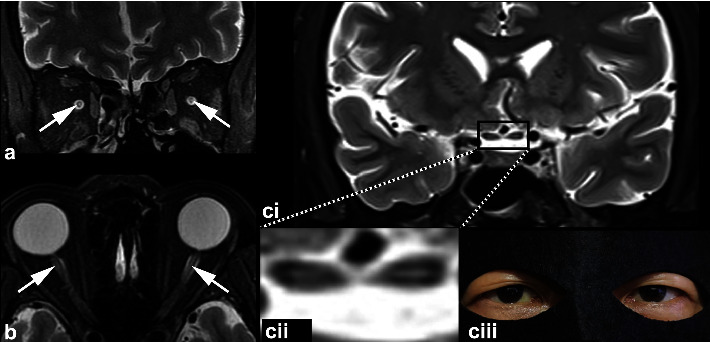
MRI identified characteristic central hyperintense T2 signals in patients with methanol-induced optic neuropathy. (a) Coronal T2 image of patient 3, demonstrating hyperintense signals (arrows) in both orbital optic nerves as “target sign.” (b) Axial T2 image of patient 6, demonstrating hyperintense signals (arrows) in both orbital optic nerves as “neutral axis sign.” (c) (i, ii) Coronal T2 image of patient 4, demonstrating hyperintense signals in both cranial optic nerves as “mask sign” (iii).

**Figure 3:**
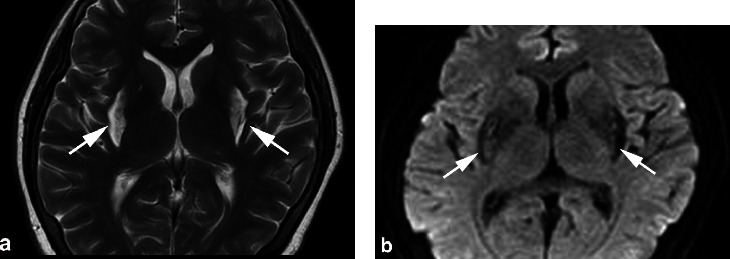
MRI identified putaminal lesions in patients with methanol-induced optic neuropathy. Brain MRI images of patient 7, 116 days after acute onset of methanol poisoning, (a) axial T2 image demonstrating hyperintense signals (arrows) in bilateral putamina, and (b) DWI b = 1000 image demonstrating dominant low signal and partial high signal in the putaminal lesion.

Table 3 shows the features of the optic nerve and brain MRI. Among the patients, 87.5% (7/8) had an abnormal optic nerve MRI, including 75.0% (6/8) T1 enhancement (Figures 1(a) and 1(b)) and 75.0% (6/8) hyperintense T2 (Figure 1(c)). Among the seven optic nerve MRI-positive patients, the orbital part was 100% (7/7) involved, and the intracranial part was 42.8% (3/7) involved. Furthermore, 71.4% (5/7) of the patients demonstrated “central” hyperintense T2 signals in the orbital segment, which presented as a “target sign” (Figure 2(a)) in the coronal scan and as a “neutral axis sign” (Figure 2(b)) in the axial scan. In the coronal scan, hyperintense T2 in the intracranial part presented as a “mask sign” (Figure 2(c) (i, ii, iii)). A putaminal lesion was identified in two patients in the brain MRIs (Figure 3).

**Figure 4:**
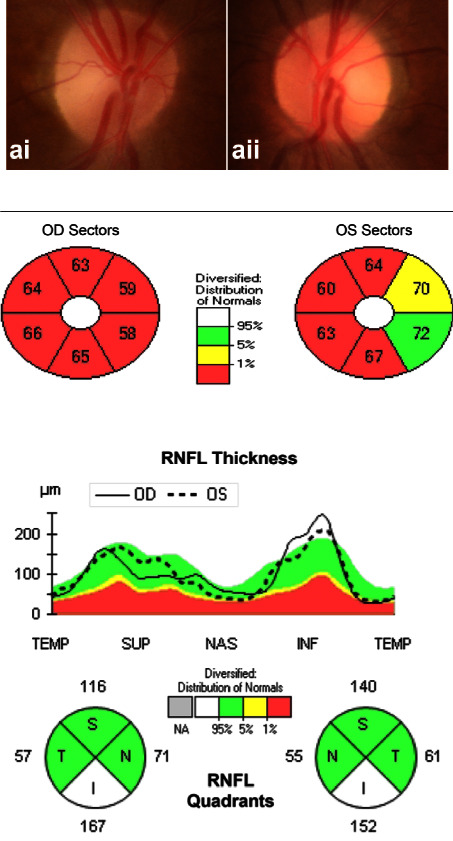
The pRNFL slightly increased, but mGCL-IPL decreased early in methanol-induced optic neuropathy. (a) (i, ii) the optic nerve is normal in the fundus photograph. (b) OCT identified obvious mGCL + IPL thinning. (c) OCT identified mild pRNFL thickening.

**Figure 5:**
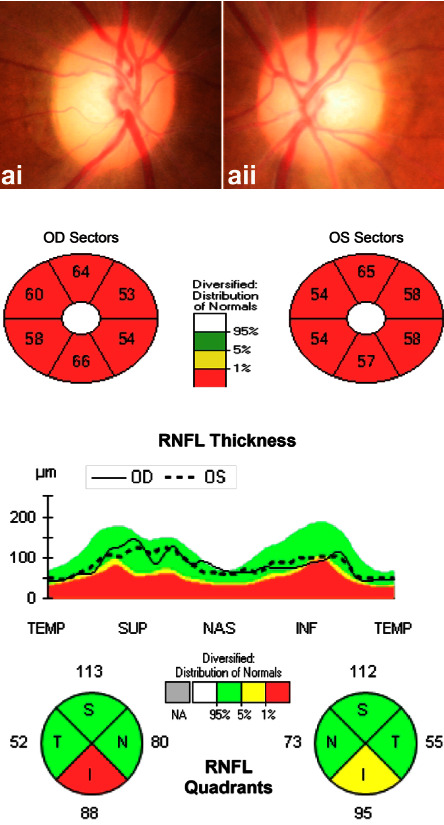
As the disease progressed, pRNFL decreased, and mGCL-IPL thinning became more severe. (a) (i, ii) the optic nerve is relatively normal in the fundus photograph. (b) OCT identified severe mGCL + IPL thinning. (c) OCT identified mild pRNFL thinning.

**Figure 6:**
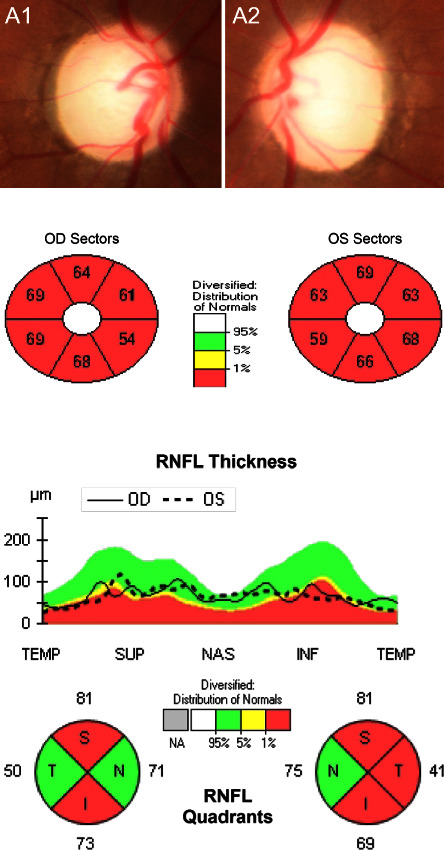
Obvious mGCL + IPL thinning, pRNFL thinning, and optic disc pallor were found in the late course of methanol-induced optic neuropathy. (a) The pale optic nerve is found in the fundus photograph. (b) OCT identified severe mGCL + IPL thinning. (c) OCT identified severe pRNFL thinning.

OCT identified an apparent decrease in the combined measurement of the thickness of the macular ganglion cell and inner plexiform layer (mGCL-IPL) for all patients. The peripapillary retinal nerve fiber layer (pRNFL) thickness increased in the early stage in one patient (Figure 4) and decreased in all patients afterward (Figures 5 and 6). The optic disc seemed normal early and became pale later.

### 3.4. Visual Function and Treatment

Visual acuity and treatments are listed in Table 4. In our study, all patients experienced bilateral, painless, acute, and dramatic visual loss regardless of the exposure route and the severity of systemic symptoms. All patients had a severe visual loss, including 87.5% (7/8) in NLP and 12.5% (1/8) in LP. The worst visual acuity was reached within two days in 87.5% (7/8) of the patients and on day 10 in 12.5% (1/8).

The patients received multiple treatments. In addition to dialysis for two patients in the emergency department, all patients received high-dose intravenous corticosteroid and nutritional therapy, including vitamin B12, idebenone, folate, and coenzyme *Q*_10_. Three patients received plasmapheresis (also called plasma exchange), one received intravenous erythropoietin, and five received hyperbaric oxygen therapy.

Among the sample, 87.5% (7/8) experienced transient and subtle visual improvement after treatment, but they all ended up having LP or NLP during their last visit. Patient 8, who experienced no visual improvement, carried the mitochondrial DNA mutation m.14502 T > C. She maintained NLP from the onset to the last visit.

Average, standard deviation, lowest, highest, frequency, and ratio values were used in the descriptive statistics of the data.

## 4. Discussion

This study evaluated the clinical characteristics of methanol-induced optic neuropathy in view of different exposure routes. The incidence of visual and systemic symptoms under different exposure routes varied in previous reports. Bebarta et al. found that methanol inhalation was a low-risk factor for visual dysfunction [28]. Givens et al. observed that many inhalational patients developed visual loss [16]. Zhonghua et al. reported that eight inhalational patients developed severe visual loss but no systemic symptoms [12]. In our study, four of five ingestion patients and all inhalation/dermal exposure patients demonstrated severe visual impairment and systemic symptoms. Thus, inhalation and dermal exposure were as effective as the oral route in producing toxic effects. The incidence and severity of impairments could be correlated more with internal exposure, such as blood levels, than with the exposure route. The onset of visual symptoms is hours to two days after ingestion [1], four days to five years after inhalation [12], and 2–10 h after dermal exposure [10, 13]. In our study, low-dose dermal exposure for 10 years also led to acute onset. Therefore, ingestion exposure always leads to an acute onset, while inhalation or dermal exposure may have a long latency (chronic course) before the acute onset. We consider that the difference lies in internal exposure, which is affected by variables such as form (liquid or vapor), concentration, dose, exposure time, and size of the exposure area.

One of the notable findings in our study was the MRI imaging feature. The MRI identified a T1 enhancement or a long T2 in the optic nerve in 87.5% of the patients. The retrobulbar and the following orbital segment of the optic nerve are most often involved, which is in agreement with previous clinical reports [12, 26] and histopathological studies [29], suggesting that the retrolaminar optic nerve is selectively vulnerable to methanol poisoning. The pathogenesis is presumed to be histotoxic anoxia in a vascular watershed area (the laminar region of the optic nerve), which is the result of direct inhibition of cytochrome oxidase by formic acid, leading to ischemic changes. Additionally, the increasing pressure following oedema in the visual pathway might further aggravate the deterioration due to ischemic changes. Different from other ischemic optic neuropathy such as hyaluronic acid obstruction-induced ischemic optic neuropathy [30], which shows no contrast enhancement in the T1-weighted image, the methanol-induced optic neuropathy shows a T1 enhancement (Figures 1(a) and 1(b)). Since it is a “metabolite ischemia” due to the action of formic acid inhibiting the cytochrome oxidase, the blood supply is not obstructed.

Furthermore, we reported for the first time the “central” hyperintense T2 signal in methanol-induced optic neuropathy. “Central” hyperintensity (Figure 2) is different from diffused or segmental hyperintensity, which has been reported in many publications (also reported in this paper in Figures 1(a)–1(c)). “Central” hyperintense means that the hyperintensity signal is specifically located in the central axis of the optic nerve. As demonstrated in Figure 2, it presented as a “target sign” in the coronal orbital optic nerve, a “neutral axis sign” in the axial orbital optic nerve, and a “mask sign” in the coronal cranial optic nerve. It was identified in 62.5% of the patients in our study. A similar feature has been reported in Leber's hereditary optic neuropathy (LHON) [31, 32]. According to our clinical observations, it usually presents in mitochondrial optic neuropathies, such as LHON and nutritional optic neuropathy, and accidentally presents in other optic neuropathies, such as radiation-induced optic neuropathy and optic neuritis. We found that the “central” hyperintense T2 lesion was highly suggestive of, although not specific to, mitochondrial optic neuropathy. We presume that it reflects the selective loss of small-caliber fibers, the P-cell population, which are disproportionately present in the papillomacular bundle and pass through the center of the optic nerve. These fibers subserve the visual functions of visual acuity, color vision, and high spatial frequency contrast sensitivity [33], which are consistent with the clinical features of dyschromatopsia and cecocentral scotoma in mitochondrial optic neuropathy. Further clinical investigation with a larger sample size may provide more detailed information, especially with a 7.0 T MRI. The brain MRI identified a long T2 in the basal ganglia in 25.0% of the patients. Interestingly, these damages are also found in Leber's “plus,” mitochondrial encephalomyopathy, lactic acidosis and stroke-like episodes (MELAS) syndrome, and Leigh syndrome, which share the same pathogenic feature as methanol-induced optic neuropathy as a mitochondrial dysfunctional disease [34].

OCT is a valuable tool for detecting ganglion cell alterations in the optic nerve disease [35, 36]. In our previous studies, we reported that the total macular thickness loss was a potential predicting factor in ethambutol-induced optic neuropathy [37] and started thinning before pRNFL in LHON [38]. In the present study, we found that the pRNFL could slightly increase in the early stage of methanol-induced optic neuropathy and decrease afterward, but the mGCL-IPL decreased dramatically in the early stage (Figures 4–6). Therefore, the mGCL-IPL is more sensitive than the pRNFL in early methanol-induced optic neuropathy. Mild optic disc edema has been previously reported in methanol-induced optic neuropathy [12]. We considered axon swelling to be induced by an axoplasmic flow disorder because of mitochondrial dysfunction.

The visual prognosis of methanol-induced optic neuropathy is controversial. According to one study, if a patient survived, most ocular symptoms would improve or resolve [39]. Another study reported high visual morbidity, which was underestimated on discharge [40]. In our study, all patients suffered from severe visual impairment, which may transiently and slightly improve after various treatments but deteriorated afterward. The co-effect of mitochondrial gene mutation may explain one patient's suffering from NLP without remission in our study.

This cohort study has some limitations. First, we used the Snellen instead of the Early Treatment Diabetic Retinopathy Study (ETDRS) visual chart in the visual acuity test, which could lead to a certain bias in low vision evaluation. Second, the small sample size led to a deviation in the assessment of the visual prognosis. Prospective studies with a larger sample size and a more extended follow-up period are warranted to confirm our findings and investigate the role of malnutrition in methanol-induced optic neuropathy.

## 5. Conclusions

Methanol-induced optic neuropathy is an acquired mitochondrial optic neuropathy with a poor visual prognosis. The high positive rates of optic nerve MRI and central hyperintense T2 signals are valuable in the recognition of methanol-induced optic neuropathy. The thinning of the mGCL-IPL is more sensitive than that of the pRNFL for early diagnosis. A mitochondrial genetic defect may be a predisposing factor for it.

## Figures and Tables

**Table 1:** Clinical characteristics of eight patients.

Patient no./age range^∗^/sex	Exposure route	Toxicant	Latency^†^	Admission time (day)^‡^	m-DNA test
1/60s/M	Ingestion	Industry alcohol	20 hours	85	N/A
2/40s/M	Ingestion	Industry alcohol	7 hours	40	N/A
3/50s/M	Ingestion	“Spirits”	20 hours	38	N/A
4/30s/M	Ingestion	“Spirits”	6 hours	18	N/A
5/50s/M	Ingestion	“Spirits”	34 hours	28	Negative
6/40s/F	Inhalation	Solid alcohol	4.3 months	43	Negative
7/40s/F	Inhalation	Industry alcohol	4.0 months	109	Negative
8/40s/F	Percutaneous	Industry alcohol	10 years	88	m.14502 *t* > *c*

M = male, F = female, and N/A = not available. ^∗^The age ranges were used instead of the exact ages according to the anonymization standards. ^†^Time from exposure to onset. ^**‡**^Time from onset to be admitted to the Neuro-Ophthalmology Department.

**Table 2:** Clinical feature and treatment in acute phase.

Patient no./age range^∗^/sex	Exposure route	Consciousness disturbance	Coma	Metabolic acidosis	Other symptoms	Dialysis
1/60S/M	Ingestion	No	No	N/A	No	No
2/40S/M	Ingestion	No	No	Yes	Cardiopulmonary	No
3/50S/M	Ingestion	Yes	Yes	Yes	Gastrointestinal/renal	Yes
4/30S/M	Ingestion	Yes	No	Yes	Gastrointestinal/renal/audial	No
5/50S/M	Ingestion	No	No	Yes	Gastrointestinal/renal	No
6/40S/F	Inhalation	No	No	N/A	Gastrointestinal	No
7/40S/F	Inhalation	Yes	Yes	Yes	Cardiopulmonary/renal	Yes
8/40S/F	Percutaneous	Yes	No	Yes	Cardiopulmonary	No

M = male, F = female, and N/A = not available. ^∗^The age ranges were used instead of the exact ages according to the anonymization standards.

**Table 3:** Characteristics of optic nerve MRI and brain MRI.

Patient no./age range^∗^/sex	Optic nerve MRI		Brain MRI
	Lesion signal	Portion	Putaminal lesion
1/60S/M	T1 + C	Negative	Negative
	Long T2	Negative	

2/40S/M	T1 + C	Retrobulbar/orbital	N/A
	Long T2	Negative	

3/50S/M	T1 + C	Retrobulbar/orbital	N/A
	Long T2	Retrobulbar/orbital^†^	

4/30S/M	T1 + C	Retrobulbar/orbital/canal	Intense T2 flair
	Long T2	Retrobulbar/orbital^†^/intracranial^†^	

5/50S/M	T1 + C	Retrobulbar/orbital	Negative
	Long T2	Retrobulbar/orbital^†^/intracranial^†^	

6/40S/F	T1 + C	Retrobulbar/orbital	Negative
	Long T2	Retrobulbar/orbital^†^	

7/40S/F	T1 + C	Negative	T1 enhancement, intense T2
	Long T2	Retrobulbar/orbital^†^/intracranial^†^	

8/40S/F	T1+C	Retrobulbar/orbital	Negative
	Long T2	Retrobulbar/orbital	

M = male, F = female, N/A = not available, and T1 + C = T1 enhancement. ^∗^The age ranges were used instead of the exact ages according to the anonymization standards. ^†^Central hyperintense signal in T2.

**Table 4:** Visual acuity and treatments of eight patients.

Patient no./age range^∗^/sex	Worst BCVA in acute phase		BCVA outcome		Transient BCVA improvement after treatment			Treatment^†^
	BCVA	Time (after onset)	BCVA (OD/OS)	Follow-up (months)	BCVA	Time (day after onset)	Treatment	
1/60S/M	NLP	3 h	NLP/NLP	42.6	FC/0.1	15	Corticosteroid	Corticosteroid
2/40S/M	NLP	1 d	NLP/NLP	39.8	FC/HM	51	Corticosteroid	Corticosteroid/HBOT
3/50S/M	NLP	2 d	NLP/NLP	38.9	FC/HM	49	Nutrition	Dialysis/corticosteroid
4/30S/M	LP	10 d	LP/LP	13.0	FC/FC	30	Corticosteroid	Corticosteroid/HBOT/PE
5/50S/M	NLP	12 h	LP/NLP	8.6	LP/FC	40	PE	Corticosteroid/HBOT/PE
6/40S/F	NLP	2 d	NLP/NLP	19.8	FC/FC	21	EPO	Corticosteroid/HBOT/EPO
7/40S/F	NLP	2 d	NLP/NLP	28.3	HM/HM	25	Corticosteroid	Dialysis/corticosteroid
8/40S/F	NLP	12 h	NLP/NLP	10.2	No improvement			Corticosteroid/HBOT/PE

M = male, F = female, BCVA = best corrected visual acuity, OD = right eye, OS = left eye, NLP = no light perception, LP = light perception, FC = finger count, HM = hand motion, HBOT = hyperbaric oxygen therapy, PE = plasmapheresis, also named plasma exchange, and EPO = erythropoietin. ^∗^The age ranges were used instead of the exact ages according to the anonymization standards. ^†^All treatments received before or during hospitalization are stated in time sequence. All patients received nutritional therapies, which were not listed here.

## Data Availability

The data involved in this study are owned by the Department of Ophthalmology of Chinese PLA General Hospital. The data are available from Dr. Huanfen Zhou (zhouzhoueye@163.com).
